# Seeking and reaching emergency care: A cross sectional household survey across two Liberian counties

**DOI:** 10.1371/journal.pgph.0002629

**Published:** 2023-11-20

**Authors:** Madeline E. Ross, Antoinette H. Wright, Mark Luke, Abraham Tamba, Heounohu Romello Hessou, Stephen Kanneh, Kumeinu Da-Tokpah, Corey B. Bills

**Affiliations:** 1 Department of Emergency Medicine, University of Colorado, School of Medicine, Aurora, CO, United States of America; 2 University of Liberia, School of Public Health, Monrovia, Liberia; 3 Emergency Medical Response-EMS/Respiratory Division, Ministry of Health, Monrovia, Liberia; 4 Stellenbosch University, Department of Emergency Medicine, Cape Town, South Africa; 5 Restore Hope Liberia, Pago Island, Oldest Congo Town, Monrovia, Liberia; University of Pennsylvania Perelman School of Medicine, UNITED STATES

## Abstract

The overwhelming burden of morbidity and mortality from injury and medical conditions requiring acute care are borne by low- and middle-income countries lacking accessible, quality care systems. Current evidence suggests the lack of prehospital care systems likely contributes to this disproportionate burden. As an initial step in a longitudinal, collaborative effort to strengthen the chain of survival for emergency conditions in Liberia, baseline attitudes and behaviors in accessing and utilizing emergency care were characterized. A multistage, proportional, cluster sampling frame was employed to conduct a cross-sectional, community-based survey of 800 households across rural Lofa County and the greater capital (Monrovia) metropolitan area. The primary outcome was facility-based utilization of emergency care within the 12 months prior to survey administration. 43.9% of individuals surveyed reported a visit to an emergency unit in the last year. Multivariable logistic regression revealed increased adjusted odds of facility-based emergency care utilization in households that were low-income, non-English-speaking, lacking electricity, or had a non-durable roof. Among these individuals, 23.6% had sought care from a community health worker, family/friend, clinic, pharmacy, or traditional healer prior. The majority of persons seeking care do so without ambulance services. 34.8% of all households have called a community member for a medical emergency, but 88.9% of survey respondents report no first aid training and cite barriers to rendering aid. This represents the first household survey to assess the perceptions and utilization of emergency care in Liberia. Formal pre-hospital care provision is limited and substantial barriers to emergency care access exist. First aid training and acceptance is lacking, despite frequent reliance on community-based aid during emergencies.

## Introduction

The overwhelming burden of morbidity and mortality from injury and other acute medical conditions are borne by low- and middle-income countries (LMICs) that lack accessible, quality emergency care systems. Though low-income countries bear nearly triple the burden of emergency conditions in high-income countries, the rate of emergency care utilization has been cited as less than 3% that of high-income countries [1]. In sub-Saharan Africa only 16 of 48 countries met the expert consensus benchmark of 80% of the population living within two hours travel time of the nearest hospital facility [2]. The lack of accessible, timely emergency and prehospital care systems in the region likely exacerbates poor patient outcomes [3].

Recent global consensus resolutions have highlighted the need for emergency and acute care [4]. The World Health Organization’s (WHO) “Emergency Care Systems Framework” emphasizes both facility-based and out of hospital emergency care as critical elements in the chain of survival [5]. Access to high quality emergency care can also be described in three phases: seeking, reaching, and receiving care. Seeking care emphasizes patient, bystander, or caregiver recognition of high-risk conditions necessitating emergency care, while the “reaching care” phase highlights mobilization to access care [6]. The success of both can be improved through systems of prehospital care.

Studies suggest significant gaps in both persons’ knowledge of and ability to recognize “danger signs” for emergency conditions in LMICs. Other barriers to emergency care access include limited infrastructure (poor roads or road safety), communication deficits, transportation challenges, and deficiencies of trained first responder personnel [7, 8].

Liberia, as is the case in many low- and middle-income country (LMIC) contexts, lacks clear national out of hospital care systems [9]. Since 2003, after decades of intermittent armed conflict, the Liberian health system has pursued a significant process of rebuilding [10]. The period of conflict led to damage of infrastructure crucial to health, a shortage of health workers, and significant poverty [11, 12]. Modest gains in systems of emergency care were further strained during the outbreak of Ebola from 2014 to 2016 [13], though recent efforts have been reignited by a national priority-setting Emergency Care Systems Assessment conducted in 2022. Currently, life expectancy at birth in Liberia is 61 years, under five mortality is 93 per 1000 live births, and rates of maternal mortality remain high (742 per 100,000 live births) [14, 15]. While out of hospital care exists in parts of the capital city of Monrovia, in most of the country prehospital care systems remain non-existent—with patients relying on largely informal mechanisms for healthcare referral and access to quality care [9]. A shortage of emergency care workers also hampers emergency care quality, though recent efforts aimed at training health workers in basic emergency care does exist [16].

One proposed mechanism to decrease barriers to accessing emergency care in the seeking and reaching phases has been to support community or lay person first aid training. Community members trained in basic emergency care have been listed as an integral component of the WHO’s “Model of Emergency Care” [6]. However questions around best practices of implementation, impact, utility, and sustainability of community-based, layperson first aid responder networks remain.

As medical sociologist David Mechanic theorized, “The probability of utilising services depends on the balance between individuals’ perceptions of their needs and their attitudes, beliefs and previous experiences with health services” [17]. In anticipation of future emergency care systems strengthening efforts in Liberia, including layperson first aid training, community-based perceptions and attitudes regarding emergency care were characterized in both rural and urban contexts.

## Methods

### Ethics statement

Ethics approval was obtained from the University of Colorado and University of Liberia Atlantic Center for Research and Evaluation (ACRE) Institutional Review Boards. Consent among study participants was obtained verbally in the individual’s preferred language.

### Study design

We performed a community-based, cross-sectional survey across two demographically and geographically diverse areas of Liberia. This study was performed in collaboration with the Emergency Medical Response-EMS/Respiratory Division of the Ministry of Health, Monrovia, Liberia, the University of Colorado, and Restore Hope Liberia.

### Study setting

The Republic of Liberia is a low-income country (by World Bank Criteria) located along the Atlantic coast in West Africa [14]. This study took place in two counties (of 15 total) in Liberia: Lofa and Montserrado. Lofa, a largely rural county, is located in northwestern Liberia, bordering Sierra Leone to the west and Guinea to the North. At the time of the study, Lofa County had an estimated population of approximately 277, 000 [18]. The second area of interest, in Montserrado County, includes both urban and peri-urban administrative areas in and surrounding the capital city of Monrovia. Monrovia and the metro area contain an estimated population of approximately 1.12 million individuals, or around 25% of the nation’s population [18].

### Sampling and sample size

Multistage cluster sampling was used to randomly select households in each of the two geographic areas under study. We relied on known county, district, and administrative mapping from previous 2008 National Population and Housing Census (NPHC) and data from the 2019–20 Liberia Demographic and Health Survey (LDHS) to construct an initial sampling frame [18, 19]. In the NPHC, individual areas were divided into enumeration areas (EAs), roughly aligning with clan or village name.

The initial sampling frame was used to calculate the sample size in each of the two geographic zones. A total of 800 households with 400 individual households in each of the two geographic areas, was generated with 5% absolute precision and 95% CI, and an assumed 4% non-response rate based on previous sampling in Liberia [19].

In Lofa County we used a two-stage sampling frame. In the first stage, we chose 40 random clusters, proportional to district size, based on known enumeration area and village town names across the county. The second stage involved a systematic random sampling of 10 households within the cluster.

Montserrado County, consists of 17 districts, though for this study we purposively sampled only urban or peri-urban districts. Given the population density in and around the capital we performed a three-stage cluster sample. In the first stage, we randomly selected townships, again proportional to known population size. We then further randomized at the level of the enumeration area, roughly equated with individual villages or clans. In the third stage, similar to Lofa, we performed systematic random sampling of 10 households within the administrative cluster. For the purposes of this study, “Monrovia” will be used to describe the greater Monrovia region sampled, including the urban and peri-urban districts of Montserrado County.

A representative adult (18 years old and older) of each household was verbally consented, regardless of sex or home ownership, and was asked a series of questions relating to individual and household sociodemographic characteristics, health information, and climate change. All participants were recruited in November 2022.

### Survey tool

We designed a 91-item questionnaire, divided into five sections related to individual and household socio-demographics, emergency care access, and care seeking behavior. Sociodemographic and emergency care questions were largely based on previously validated questions from the Democratic Republic of Congo, South Africa, and Cameroon [20–22]. Eleven additional questions, not explored in this study, specific to climate related health effects, were also based on previously validated studies in other low-income countries [23].

In total, 16 research assistants (RA) were trained. Given the large number of languages in Liberia, the survey was written in English and not translated to additional languages. In all cases, the survey was administered verbally by the RAs but interpreted to the respondents preferred local language. Research assistants underwent a 2-day focused training, including piloting the tool among each other and among sample households to clarify consistency in administration and responses—regardless of the language used—prior to full study administration.

### Patient and public involvement

Initial focus groups with the public, including key informant community members, members of the health ministry, and prehospital system employees and volunteers were involved in the piloting and design of some survey questions. Patients were not involved in the design, nor were they specifically targeted as respondents to the survey.

### Data analysis

Initial survey data was transcribed, with collected forms entered, cleaned, and de-identified into Excel prior to analysis performed via Stata (Version 14.2, College Station, TX). Initial respondent and household demography were presented as number and percents and averages and standard deviations as appropriate. Demographic, social and health related data was stratified by geographic area. Emergency care utilization in the last 12 months related to key demographics social and health-related variables were compared using chi-square analysis for categorical variables (or Fisher’s exact test when appropriate).

Univariable logistic regression was used to assess individual predictor variables with the primary outcome of interest. Multiple multivariable logistic regression models were performed using combined and stratified data by geographic region. Predictor variables were based on prior methodologic theory and were included if the p-value was <0.10 in univariable analysis, variables thought to be known historical risk factors, and confounders of emergency care utilization in the past year [24].

The number of variables used in the final models differed between combined and stratified owing to the different number of individuals in each subgroup accessing emergency care in the last year. We report unadjusted and adjusted odds ratios (aORs) and 95% confidence intervals (CI) for predictor variables to the primary outcome variable. A p-value of <0.05 was considered statistically significant. In each model we report the calculated area under the curve (AUC) to assess for discrimination.

## Results

Of the 800 individuals surveyed (Table 1), a majority were male (N = 461, 57.6%) with a mean age of 40.62 years (SD 14.72). While half of the total households surveyed spoke English as the primary language at home, the results varied significantly between counties with more than 90% of households speaking English in the greater Monrovia region, but more than 90% of households speaking a language other than English in Lofa County. Over half of respondents in Lofa county reported no prior school-based education and 39.4% were not literate with 66.3% employed in farming. In contrast, over half of participants in the greater Monrovia region had a secondary or higher school-based education with high rates of literacy and employment in non-farming occupations. Notably, 87.5% of urban and peri-urban respondents had access to a phone, an important resource for emergency system activation, while less than half of rural Lofa participants had one.

**Table 1:** Baseline demographic characteristics among household respondents in two geographic regions of Liberia.

Characteristic	Total	Monrovia Area	Lofa County
	N = 800	N = 399	N = 401
Sex (%)			
Female	337 (42.1)	123 (30.8)	214 (53.4)
Male	461 (57.6)	276 (69.2)	185 (46.1)
Mean Age (SD)	40.62 (14.72)	39.34 (14.35)	41.96 (14.99)
Age (%)			
18–29	181 (22.6)	107 (26.8)	74 (18.5)
30–39	235 (29.4)	120 (30.1)	115 (28.7)
40–49	141 (17.6)	66 (16.5)	75 (18.7)
50–59	95 (11.9)	46 (11.5)	49 (12.2)
60+	94 (11.8)	43 (10.8)	51 (12.7)
Primary language (%)			
English	422 (52.8)	325 (81.5)	119 (29.7)
Other	377 (47.1)	74 (18.5)	282 (70.3)
Primary household language (%)			
English	396 (49.5)	370 (92.7)	26 (6.5)
Other	404 (50.5)	29 (7.3)	375 (93.5)
Education (%)			
None	311 (38.9)	86 (21.6)	225 (56.1)
Primary	90 (22.5)	45 (11.3)	45 (11.2)
Secondary	213 (26.6)	148 (37.1)	65 (16.2)
College/University	90 (22.5)	70 (17.5)	20 (5.0)
Graduate	84 (10.5)	47 (11.8)	37 (9.2)
Literacy (%)			
No	320 (40.0)	79 (19.8)	241 (60.1)
Yes	476 (59.5)	318 (79.7)	158 (39.4)
Occupation (%)			
Unemployed	93 (11.6)	84 (21.1)	9 (2.2)
Farmer	269 (33.6)	3 (0.8)	266 (66.3)
Business	178 (22.3)	158 (39.6)	20 (5.0)
Other	194 (24.3)	142 (35.6)	52 (13.0)
Phone Access (%)			
No	287 (35.9)	50 (12.5)	237 (59.1)
Yes	513 (64.1)	349 (87.5)	164 (40.9)
Smartphone (%, N = 513)			
No	268 (52.2)	149 (42.7)	119 (72.6)
Yes	244 (47.6)	201 (57.6)	43 (26.2)

The characteristics of households surveyed also varied between geographic regions sampled (Table 2). An average of approximately seven persons (6.93, SD 4.55) occupied households surveyed with approximately three of those persons (2.91, SD 2.23) under the age of 18 years. Less than two occupants per household were reported to generate income (1.54, SD 1.35). Of the respondents who answered questions about income, 53% of households in Monrovia and 79% of households in Lofa County lived on less than 100 USD per month. Households in Lofa County were also less likely to report homes constructed from durable materials with latrine access or electricity. The majority of respondents in both counties reported adequate access to water for drinking and cleaning, though more households treat their drinking water with chlorine, boiling, or filtering in the communities in and around Monrovia than in in Lofa County. Cooking fuel source was also overwhelmingly wood or charcoal in both counties. However, the majority of respondents in Lofa County report cooking outdoors, while the cooking location is more variable in Monrovia.

**Table 2:** Household characteristics among households in two geographic regions of Liberia.

Characteristic	Total	Monrovia Area	Lofa County
	N = 800	N = 399	N = 401
Mean occupants (SD)	6.93 (4.55)	7.82 (5.66)	6.06 (2.80)
Mean occupants <18 (SD)	2.91 (2.23)	3.02 (2.61)	2.79 (1.74)
Mean occupants with income (SD)	1.54 (1.35)	1.76 (1.28)	1.24 (1.38)
Household monthly income (%)			
<100 USD	215 (26.9)	94 (23.6)	121 (30.2)
≥100 USD	115 (14.4)	83 (20.8)	32 (8.0)
Dwelling standards (%)			
Durable walls	452 (56.5)	351 (88.0)	101 (25.2)
Durable roof	690 (86.3)	394 (98.7)	296 (73.8)
Durable floor	456 (57.0)	353 (88.5)	103 (25.7)
Latrine access (%)			
No	211 (26.4)	7 (1.8)	204 (50.9)
Yes	586 (73.3)	391 (98.0)	195 (48.6)
Adequate water access for drinking (%)			
No	46 (5.8)	11 (2.8)	35 (8.7)
Yes: community pump	428 (53.5)	213 (53.4)	215 (53.6)
Yes: purchase	139 (17.4)	121 (30.3)	18 (4.5)
Yes: running water/tap	184 (23.0)	54 (13.5)	130 (32.4)
Treatment of drinking water (%)			
No	493 (61.6)	171 (42.9)	322 (80.3)
Yes*	284 (35.5)	217 (54.4)	67 (16.7)
Adequate water for cleaning (%)			
No	216 (27.0)	88 (22.1)	128 (31.9)
Yes	574 (71.8)	309 (77.4)	265 (66.1)
Water access for agriculture (%)			
No	462 (57.8)	300 (75.2)	162 (40.4)
Yes	334 (41.8)	97 (24.3)	237 (59.1)
Soap access for hygiene (%)			
No	322 (40.2)	135 (33.8)	187 (46.6)
Yes	471 (58.9)	263 (65.9)	208 (51.9)
Household electricity (%)			
No	417 (52.1)	70 (17.5)	347 (86.5)
Yes	379 (47.4)	327 (82.0)	52 (13.0)
Cooking location (%)			
Outdoor	584 (73.0)	227 (56.9)	357 (89.0)
Indoor	207 (25.9)	167 (41.9)	40 (10.0)
Cooking fuel source (%)			
Charcoal or wood	767 (95.9)	376 (94.2)	391 (97.5)
Gas or electric	29 (3.6)	23 (5.8)	6 (1.5)

*chlorine, boil or filter

Less than 4% of all individuals surveyed reported having health insurance (Table 3). Though less than half of all respondents reported having a community health assistant in their community, more individuals in Lofa County endorsed having this community resource (N = 170, 42.4% in Lofa County vs N = 144, 36.1% in Monrovia). Of note, community health assistants are trained, compensated individuals that live and work in communities across Liberia to provide an integrated and standardized service delivery package, focusing on health promotion and epidemiologic surveillance for households located 5 kilometers from the nearest health facility [25]. Overall, the rates of emergency and routine health visits per household per month were similar in both regions. Additionally, the destinations (clinic vs hospital or health center) at which households sought emergency care did not differ significantly between day and night times. However, the mechanism of transport by which households sought emergency care varied between regions. During the daytime, the majority of households in both areas travel via motorbike, keke (a 3-wheeled motorbike), or car (63.2% in Monrovia, 55.1% in Lofa) for emergency visits, but nearly a quarter of households in Lofa County report walking (24.9%) compared to only 8.3% in the greater Monrovia region. The average cost of transport during the daytime was found to be $5.08 USD (SD 7.92). At night, fewer households walked in both areas with higher average costs of vehicular transport ($7.55 USD, SD 8.78) and no significant increase in ambulance usage.

**Table 3:** Household healthcare resources and utilization.

Question	Total	Monrovia Area	Lofa County
	N = 800	N = 399	N = 401
Health Insurance (%)			
No	741 (92.6)	268 (92.2)	373 (93.0)
Yes	29 (3.6)	20 (5.0)	9 (2.2)
Community Health Assistant in Community (%)			
No	459 (57.4)	247 (61.9)	212 (52.9)
Yes	314 (39.3)	144 (36.1)	170 (42.4)
Mean household routine health visits per year (SD)	3.20 (3.22)	2.43 (2.66)	3.97 (3.53)
Mean household emergency health visits per year (SD)	0.90 (1.48)	0.79 (1.56)	1.04 (1.37)
Emergency health visit location, day (%)			
Clinic	361 (45.1)	173 (43.4)	188 (46.9)
Health center or hospital	255 (31.9)	112 (28.1)	143 (35.7)
Other	8 (1.0)	6 (1.5)	2 (0.5)
Emergency health visit location, night (%)			
Clinic	317 (39.6)	142 (35.6)	175 (43.6)
Health center or hospital	266 (33.3)	130 (32.6)	136 (33.9)
Other	3 (0.4)	2 (0.5)	1 (0.2)
Emergency health visit transport, day (%)			
Walk or hammock	133 (16.6)	33 (8.3)	100 (24.9)
Ambulance	15 (1.9)	2 (0.5)	13 (3.2)
Motorbike, car, keke	473 (59.1)	252 (63.2)	221 (55.1)
Emergency health visit transport, night (%)			
Walk or hammock	38 (4.8)	17 (4.3)	21 (5.2)
Ambulance	15 (1.9)	1 (0.3)	14 (3.5)
Motorbike, car, keke	482 (60.3)	252 (63.2)	230 (57.4)
Emergency health visit transport cost, day in USD (SD)	5.08 (7.92)	4.84 (7.68)	5.28 (8.14)
Emergency health visit transport cost, night in USD (SD)	7.55 (8.78)	6.44 (7.93)	8.52 (9.36)
Ambulance ever used for health emergency occurring in household (%)			
No	707 (88.4)	371 (93.0)	336 (83.8)
Yes	82 (10.3)	27 (6.8)	55 (13.7)
Ambulance considered, but not used, reason (%, N = 107)			
Cost	14 (13.1)	5 (6.0)	9 (39.1)
Unsure how to activate	4 (3.7)	3 (3.6)	1 (4.3)
Ambulance broke down	21 (19.6)	16 (19.0)	5 (21.7)
Ambulance refused to transport	6 (5.6)	5 (6.0)	1 (4.3)
Ambulance never arrived	7 (6.5)	2 (2.4)	5 (21.7)
Other	55 (51.4)	53 (63.1)	2 (8.7)
Ambulance phone number (%)			
Phone number does not exist	57 (7.1)	3 (0.8)	54 (13.5)
Phone number unknown	320 (40.0)	97 (24.3)	83 (20.7)
Phone number known	185 (23.1)	281 (70.4)	44 (11.0)
Perceived cost of calling ambulance (%)			
Yes, cost exists	132 (16.5)	42 (10.5)	90 (22.4)
No, cost does not exist	426 (53.3)	287 (71.9)	139 (34.7)
Mean cost of ambulance transport in USD (SD)	21.36 (17.76)	15.12 (15.97)	25.41 (17.82)
Ambulance arrival time (%)			
Never arrived	72 (29%)	65 (40.9)	7 (8.5)
Arrived in <30 minutes	92 (38.2)	48 (30.2)	44 (53.7)
Arrived in 30–60 minutes	60 (24.9)	44 (27.7)	16 (19.5)
Arrived in >60 minutes	17 (7.1)	2 (1.3)	15 (18.3)

Only 10.3% of all households reported having ever used an ambulance for an emergency. More households in Lofa County reported ambulance usage (N = 55, 13.7%) than in urban and peri-urban households of Monrovia (N = 27, 6.8%). Of those who had used ambulances, the majority experienced ambulance arrival within 60 minutes, though nearly one third reported that ambulances never arrived (29%). Reported cost of ambulance use was variable with a mean cost of $21.36 USD (SD 17.76). Of the 800 individuals surveyed, 107 respondents endorsed that they or someone in their household had considered or called an ambulance, but ultimately did not use one. Reasons for the lack of ultimate utilization included cost (6.0% in Monrovia vs 39.1% in Lofa County), ambulance break down (19.0% in Monrovia vs. 21.7% in Lofa County), ambulance never arrived (2.4% in Monrovia vs. 21.7% in Lofa County), ambulance refused to transport (6.0% in Monrovia vs. 4.3% in Lofa County), and others.

When specifically asked about knowledge of a phone number to activate an ambulance, 40% of all persons surveyed reported not knowing the number. Though an emergency activation number does exist for the larger Monrovia area, no such number exists for prehospital system activation in Lofa County. This was reflected in the 13.5% of households in Lofa County replying that they did not believe a number to exist, as well as by the fact that over 50% of households in this area did not reply to this question. Only 0.8% of households in Monrovia reported no number to be in existence. There were similarly low response rates in Lofa County regarding the perceived cost of a call to activate an ambulance, while most respondents in Monrovia reported no cost associated with this type of call (N = 287, 71.9%).

Many more households in Lofa County had called a community member for help in a medical emergency (Table 4) occurring both in the household and in public settings (N = 232, 57.9% in home; N = 173, 43.1% in public) than in the greater Monrovia area (N = 46, 11.5% in home; N = 18, 4.5%). Community health assistants/volunteers were the most common type of community member called for these emergencies in both settings in Lofa County with family and friends next most common. Similarly, half of households in Lofa County (N = 204, 50.9%) had experienced spontaneous aid from a community member who had not been called during an emergency in public, primarily by community health assistants/volunteers, while only 6.5% reported this aid in Monrovia. Of note, 88.9% of all individuals surveyed have no prior first aid training and 75% perceived barriers to providing first aid during a health emergency. Barriers cited included concerns around lack of skills/experience, disease/illness exposure, legal retribution, and community violence.

**Table 4:** Community assistance during health emergencies.

Question	Total	Monrovia Area	Lofa County
	N = 800	N = 399	N = 401
Called community member for help in emergency occurring in household (%)			
No	486 (60.8)	347 (87.0)	139 (34.7)
Yes	278 (34.8)	46 (11.5)	232 (57.9)
If yes, who? (%)			
Community Health Assistant/Volunteer	125 (45.0)	2 (4.3)	135 (58.2)
Family/Friend	90 (32.4)	20 (43.5)	70 (30.2)
Other	173 (62.2)	24 (52.2)	27 (11.6)
Called community member for help in emergency occurring in public (%)			
No	569 (71.1)	374 (93.7)	195 (48.6)
Yes	191 (23.9)	18 (4.5)	173 (43.1)
If yes, who? (%)			
Community Health Assistant/Volunteer	133 (69.6)	2 (11.1)	131 (75.7)
Family/Friend	9 (4.7)	5 (27.8)	4 (2.3)
Other	49 (25.7)	11 (61.1)	38 (22.0)
Community member aided in emergency occurring in public without being called (%)			
No	539 (67.4)	369 (92.5)	170 (42.4)
Yes	230 (28.8)	26 (6.5)	204 (50.9)
If yes, who? (%)			
Community Health Assistant/Volunteer	122 (53.0)	1 (3.8)	111 (54.4)
Family/Friend	68 (29.6)	8 (30.8)	6 (2.9)
Other	40 (17.4)	17 (65.4)	87 (42.6)
Prior first aid training (%)			
No	711 (88.9)	375 (94.0)	336 (83.8)
Yes	52 (6.5)	23 (5.8)	29 (7.2)
Perceived barriers to providing first aid during a health emergency (%)			
No	195 (24.4)	105 (26.3)	90 (22.6)
Yes	605 (75.6)	295 (73.8)	309 (77.4)
If yes, what? (%)			
Lack of skills/experience	219 (36.2)	150 (50.8)	69 (22.3)
Disease/illness exposure	209 (34.5)	77 (26.1)	132 (42.7)
Legal retribution	132 (28.1)	55 (18.6)	77 (24.9)
Community violence	45 (7.4)	13 (4.4)	31 (10.0)

Less than half of all adults surveyed (N = 351, 43.9%) had personally sought care in an emergency unit (EU) in the 12 months prior to survey administration (Table 5). Of those that had, the overwhelming majority reported a medical concern (N = 323, 92%) as the reason for the EU visit with far less reporting a trauma or injury-related concern (N = 24, 6.8%) or a mental health concern (N = 4, 1.1%). Just over half of Monrovia-based individuals (N = 99, 51.5%) reported dissatisfaction with the care received during their EU visit compared to the less than one third of Lofa County-based individuals (N = 46, 29.0%) dissatisfied with their EU care. Only 23.6% of adults who had personally sought care from an EU in the past 12 months had sought care for their health concern elsewhere prior to the EU. However, of those who had, clinics were the most visited in Monrovia and community health assistants/volunteers the most visited in Lofa County. Care prior to an EU visit was also sought from family/friends and pharmacies with only one individual reporting first visiting a traditional healer.

**Table 5:** EU-based care.

Question	Total	Monrovia Area	Lofa County
	N = 800	N = 399	N = 401
Personally sought care in EU in past 12 months (%)			
No	432 (54.0)	205 (51.4)	227 (56.6)
Yes	351 (43.9)	192 (48.4)	159 (39.7)
If yes, for what? (%)			
Medical	323 (92.0)	185 (96.4)	138 (89.0)
Injury or Trauma	24 (6.8)	7 (3.6)	17 (11.0)
Mental Health	4 (1.1)	0 (0)	4 (2.6)
If yes, satisfied with care? (%)			
Extremely Dissatisfied	109 (31.1)	83 (43.2)	26 (16.4)
Dissatisfied	36 (10.3)	16 (8.3)	20 (12.6)
Satisfied	162 (46.2)	79 (41.1)	83 (52.2)
Very Satisfied	37 (10.5)	14 (7.3)	23 (14.5)
Extremely Satisfied	3 (0.9)	0 (0)	3 (1.9)
Sought care for emergency elsewhere prior to EU (%)			
No	265 (76.4)	161 (83.9)	104 (67.1)
Yes	82 (23.6)	31 (16.1)	51 (32.9)
If yes, where? (%)			
CHA/CHV	29 (35.4)	1 (3.2)	28 (54.9)
Family/friends	25 (30.5)	4 (12.9)	21 (41.2)
Clinic	20 (24.4)	19 (61.3)	1 (2.0)
Pharmacy	7 (8.5)	7 (22.6)	0 (0)
Traditional healer	1 (1.2)	0 (0)	1 (2.0)
Sought care for emergency elsewhere instead of health facility (%)			
No	498 (62.3)	256 (64.2)	242 (60.3)
Yes	245 (30.6)	142 (35.6)	103 (25.7)
If yes, from who? (%)			
Family/friends	89 (36.3)	47 (33.1)	42 (40.8)
Traditional healer	39 (15.9)	32 (22.5)	7 (6.8)
Community member/CHA	34 (13.9)	12 (8.5)	22 (21.4)
Other	10 (4.1)	3 (2.1)	7 (6.8)
If yes, for what? (%)			
Medical	211 (86.1)	115 (81.0)	96 (93.2)
Injury or Trauma	28 (11.4)	24 (16.9)	4 (3.9)
Mental Health	5 (2.0)	3 (2.1)	2 (1.9)
If yes, why not a health facility? (%)			
High cost	119 (48.6)	78 (54.9)	41 (39.8)
Lack of transportation	104 (42.4)	70 (49.3)	34 (33.0)
Family disapproval	82 (33.5)	70 (49.3)	12 (11.7)
Self-medication	63 (25.7)	55 (38.7)	8 (7.8)
Distance	53 (21.6)	37 (26.1)	16 (15.5)
Preference	25 (10.2)	22 (15.5)	3 (2.9)
Facility lacks resources	3 (1.2)	3 (2.1)	0 (0)

30.6% of individuals endorsed having had a complaint requiring urgent medical attention in the last 12 months for which they did not visit a health facility for diagnosis and treatment. In the communities in and around Monrovia, individuals primarily sought care from family/friends (N = 47, 33.1%) or traditional healers (N = 32, 22.5%) for medical conditions (N = 115, 81%). In Lofa County, individuals primarily sought care from family/friends (N = 42, 40.8%) and community members/CHAs (N = 22, 21.4%) also for mostly medical conditions (N = 96, 93.2). Factors noted to have impacted the choice not to seek care from a health facility for these conditions included high cost, lack of transportation, family disapproval, self-medication, distance, preference, and lack of resources at the facility.

Only 13.6% of households reported having had a death of a household member in the 12 months prior to survey administration, though 26 households (3.3%) did report multiple deaths in this period (Table 6). Of the 152 total deaths reported, the mean age at death was 46 (SD 25.8) with less than 6% of deaths occurring in household members under the age of 5 years. However, 52% of all deaths occurred in persons aged 18 to 49 years. The majority of deaths occurred at home though 64.5% of households reported that the deceased had received medical care within 24 hours of their death. Though medical causes were also the leading type of emergency condition linked to the deaths, an increasing proportion of injury or trauma-related conditions were noted as causes (N = 27, 17.8%) compared to the causes for EU visits among the living surveyed individuals.

**Table 6:** Deaths in household.

Question	Total	Monrovia Area	Lofa County
	N = 800	N = 399	N = 401
Death in household in the last 12 months (%)			
No	691 (86.4)	353 (88.5)	338 (84.3)
Yes	109 (13.6)	46 (11.5)	63 (15.7)
Total number of deaths	152	55	97
Death rate (per 1000 people)	29.22 (79.60)	22.57 (69.30)	34.99 (87.93)
Mean age at death (SD)	46.0 (25.8)	44.47 (25.22)	48.95 (26.91)
Age at death (%)			
<1 years	4 (2.6)	4 (7.3)	0 (0)
1–5 years	5 (3.3)	1 (1.8)	4 (4.1)
5–17 years	12 (7.9)	2 (3.6)	10 (10.3)
18–49 years	79 (52.0)	22 (40.0)	56 (57.7)
>50	49 (32.2)	22 (40.0)	27 (27.8)
Location of deaths (%)			
Home	69 (45.4)	27 (49.1)	42 (43.3)
Health center or hospital	48 (31.6)	18 (32.7)	30 (30.9)
Clinic	19 (12.5)	6 (10.9)	13 (13.4)
Other health facility	6 (3.9)	0 (0)	6 (6.2)
Public	9 (5.9)	4 (7.3)	5 (5.2)
Medical care within 24 hours of death (%)			
No	46 (30.3)	19 (34.5)	27 (27.8)
Yes	98 (64.5)	37 (67.3)	61 (62.9)
Perceived cause of death			
Medical	96 (63.2)	39 (70.9)	57 (58.8)
Injury or Trauma	27 (17.8)	4 (7.3)	23 (23.7)
Mental Health	6 (3.9)	1 (1.8)	5 (5.2)

Our primary outcome of interest was facility-based emergency care utilization in the 12 months prior to survey administration. Analysis revealed a primary language, geographic area, employment, income, household electricity, clean water access, latrine access, durable floor, durable roof, household death within 12 months, and perceived barriers to rendering first aid to be significantly associated with this outcome (see S1 Table).

Multivariable analysis demonstrated higher adjusted odds of having sought facility-based emergency care within the last year among individuals of households with non-durable roofs and no electricity (though there is likely significant interaction between these factors), those located in and around Monrovia, and those with a primary language other than English (Table 7). Individuals from households with no access to community health assistants, as well as those individuals who reported concerns about rendering first aid during a health emergency, also had higher adjusted odds.

**Table 7:** Univariable and multivariable characteristics associated with facility-based emergency care utilization in the 12 months prior to survey.

Characteristic	Unadjusted OR (95% CI)	Adjusted OR (95% CI)*
Non-durable roof	7.65 (4.56–12.85)	10.20 (5.33–19.52)
Monrovia	1.41 (1.07–1.87)	10.03 (5.58–18.04)
Perceived barriers to providing first aid during a health emergency	6.74 (3.59–12.67)	9.57 (4.69–19.53)
Non-English speaking household	1.90 (1.43–2.52)	2.06(1.35–3.15)
No electricity at home	1.53 (1.15–2.03)	2.23 (1.26–3.95)
No CHA	1.77 (1.32–2.38)	1.67 (1.14–2.45)
No latrine access	1.74 (1.27–2.39)	--
Household death 12 months	1.68 (1.12–2.53)	--
Unemployed	1.60 (1.03–2.48)	--
Low income (<$100USD/mo)	1.56 (1.03–2.35)	--

AUC = 0.7972

Stratified multivariable analysis among the subgroup from communities in and around Monrovia (see S2 Table) revealed perceived barriers to rendering first aid (such as concerns about legal retribution, community violence, or disease exposure) had the strongest association with the primary outcome (aOR 6.57, 95%CI: 3.33–12.96).

For the Lofa County subgroup (see S3 Table), stratified multivariable analysis revealed that the household factor of a non-durable roof had the highest adjusted odds for having sought facility-based emergency care within the last year (aOR 12.60, 95%CI: 7.03–22.56), followed by lack of electricity and household death within 12 months.

## Discussion

Our results provide significant insight into the patterns of behavior around seeking and reaching emergency care across rural, peri-urban, and urban contexts that is likely representative of the patterns across Liberia. Our findings suggest limitations in access to facility-based emergency care, as well as provision of both informal and formal out-of-hospital emergency care.

We observed very similar proportions of household emergency health visits as those described in the Democratic Republic of the Congo, with just over half of households having made one or more emergency visits to a health facility (clinic, health center, or hospital) in the last year [20]. Of note, our data revealed a high proportions of clinics as the destination for these emergency health visits in Liberia, which may not have sufficient capacities to deliver the necessary emergency care.

Compared to findings from Cameroon, where only 7% of the 34.8% of individuals experiencing an emergency condition utilized an emergency unit (instead utilizing outpatient clinics primarily), significantly increased rates of emergency unit utilization were reported among individuals surveyed in Liberia, with nearly 44% reporting a visit to an emergency unit in the last 12 months [21]. Even so, nearly a third of individuals in this study reported having had a medical condition requiring urgent or emergent medical attention for which they did not seek care from a health facility. Though approximately 10% of these individuals cited this to be a preference, the two most frequent factors influencing the lack of use were high cost and lack of transportation, suggesting significant unmet emergency care needs across the spectrum of access. In both Cameroon and the DRC, economic concerns and self-medication were also found to be among the most frequent reasons for not seeking healthcare [20, 21].

Estimated cost of transportation for an emergency health visit ranged from $5.08–7.55 USD, which may represent a large financial burden when 65% of all those who responded to income questions reported living on less than $100 USD per month. Ambulance services were also perceived to have prohibitive costs. However, low rates of ambulance use are likely tied to a general lack of availability compounded by cost, activation challenges, and other factors. As Liberia’s healthcare system is largely financed by households and external development partners in a fee-for-service delivery model with only 4% of the government’s general expenditure allotted to health, cost is further incurred upon arrival to an emergency unit or other health facility [26, 27].

Interestingly, our analysis revealed higher adjusted odds of having sought facility-based emergency care within the last year among individuals from households with non-durable roofs or no electricity in contrast to country level trends suggesting lowest rates of emergency care utilization among low-income subgroups [1]. Surveyed households with a monthly income over $100 USD did have significantly higher rates of routine healthcare usage compared to low-income households (p-value 0.044, though notably both rates were above 90%). It is possible this differential access to or utilization of routine care may have contributed to the higher rates of low-income household utilization of emergency care: a well-evidenced relationship that has been documented in a variety of other contexts [28].

Beyond financial barriers, lack of the appropriate skills mix in the health workforce critically impacts emergency care delivery. The most recent 2018 estimates suggest the density of health workers remains extremely low throughout Liberia: medical doctors at 0.1 per 1,000 persons and nursing/midwives at 2 per 1,000 persons [14]. Given the relative paucity of availability of health workers, geographic distance to health facilities disproportionately affects access for many rural populations with over 1 million Liberians living more than an hour walk from a health facility [25]. This may explain why individuals from households located in and around Monrovia had higher adjusted odds of having sought facility-based emergency are within the last year. And contrastingly, this may also explain why individuals from households in Lofa County were more likely to report having sought care from someone or somewhere else *prior* to visiting a health facility.

Our findings demonstrate substantial proportions of individuals in both regions have called for or received care from community members during a health emergency. A large proportion of these community members were reported to be Community Health Assistants (CHA) or general Community Health Volunteers (gCHV), particularly in Lofa County. The robust network of CHAs formalized within Liberia over the last ten years has been crucial in improving access to essential primary health care services and reach of epidemic surveillance [25, 29]. This survey suggests that although not trained as emergency care providers, CHAs appear to be serving not uncommonly in this de facto role in the out-of-hospital setting.

Family and friends are also frequently called upon during health emergencies, yet the vast majority of individuals surveyed have no prior training in first aid and cite other critical concerns regarding rendering it beyond a “lack of skills”. Likely rooted in the national experience with the Ebola outbreak, concerns about disease or illness exposure during first aid provision were reported. Fear of legal retribution is not unfounded, as Liberia’s legal frameworks do have language promoting universal access to emergency care, but no true bystander protections exist for individuals who may provide first aid [30]. Among individuals from households in Monrovia, these perceived barriers to rendering first aid were also associated with higher odds of having sought facility-based emergency care within the past year. This raises the question, though unasked in this survey, of whether this attitude regarding first aid is perhaps bidirectional with fears of not only administering, but also potentially receiving it.

## Limitations

While we pursued a rigorous sampling frame, limited understanding of health-related terminology, hesitancy to discuss sensitive subjects such as finances, and recall bias may have impacted survey responses and individual question response rates. The survey was also verbally interpreted into a variety of languages by research assistants without official language certifications. No individual emergency care outcomes were analyzed. Furthermore, sociocultural patterns specific to Liberia may limit broader generalizability of the study.

## Conclusions

This project represents the first household survey to assess perceptions and utilization of emergency care in Liberia. Less than half of respondents reported utilizing facility-based emergency care in the last year. And so the question remains: was this because it was not needed, because it was not sought, or because it was not reached? Our survey results paint a mixed picture. A clearer picture remains that formal pre-hospital emergency care provision is extremely limited and many barriers to care exist in the out-of-hospital setting in these two Liberian counties. Thus, meaningful realization of the universal right to health in Liberia will require increased strengthening to all stages of the chain of survival for emergency conditions.

## Supporting information

S1 Checklist STROBE statement—checklist of items that should be included in reports of observational studies. (DOCX)

S1 Table Characteristics associated with facility-based emergency care utilization in the last 12 months. (DOCX)

S2 Table Stratified univariable and multivariable characteristics associated with facility-based emergency care utilization in the 12 months prior to survey, among residents in and around Monrovia, Montserrado County (N = 399). (DOCX)

S3 Table Stratified univariable and multivariable characteristics associated with facility-based emergency care utilization in the 12 months prior to survey, among residents of Lofa County (N = 401). (DOCX)
