# Supplementary material for: Seeking and reaching emergency care: A cross sectional household survey across two Liberian counties
Source: PLOS Glob Public Health. 2023 Nov 20;3(11):e0002629. doi: 10.1371/journal.pgph.0002629 (PMC10659191; doi:10.1371/journal.pgph.0002629)
Supplement: S1 Table — (DOCX) [file pgph.0002629.s002.docx]

S1 Table. Characteristics associated with facility-based emergency care utilization in the last 12 months.

|  | Facility-based emergency care utilization in prior 12 months | |  |
| --- | --- | --- | --- |
| Characteristic | No (%) | Yes (%) | p-value |
|  | 449 (56.1) | 351 (43.9) |  |
| Sex (%)  Male  Female | 187 (41.6)  261 (58.1 | 150 (42.7)  200 (57.0) | 0.751 |
| Age (%)  <50  >50 | 319 (71.0)  108 (24.1) | 238 (67.8)  81 (23.1) | 0.975 |
| Primary Language of English (%)  No  Yes | 177 (39.4)  272 (62.1) | 179 (51.0)  172 (49.0) | <0.001 |
| Phone Access (%)  No  Yes | 170 (37.9)  279 (62.1) | 117 (33.3)  234 (66.7) | 0.185 |
| Geographic Area (%)  Monrovia  Lofa County | 207 (46.1)  242 (53.9) | 192 (54.7)  159 (45.3) | 0.016 |
| Literacy (%)  No  Yes | 180 (40.1)  268 (59.7) | 140 (39.9)  208 (59.3) | 0.988 |
| Unemployed (%)  No  Yes | 378 (84.2)  44 (9.8) | 263 (74.9)  49 (14.0) | 0.034 |
| Electricity (%)  No  Yes | 214 (47.7)  234 (52.1) | 203 (57.8)  145 (41.3) | 0.003 |
| Cooking Location (%)  Outside  Inside | 327 (72.8)  120 (26.7) | 257 (73.2)  87 (24.8) | 0.622 |
| Income, USD (%)  <100  >100 | 104 (23.2)  75 (16.7) | 111 (31.6)  40 (11.4) | 0.003 |
| Latrine Access (%)  No  Yes | 97 (21.6)  350 (78.0) | 114 (32.5)  236 (67.2) | 0.001 |
| Clean Water Access (%)  No  Yes | 91 (20.3)  353 (78.6) | 125 (35.6)  221 (63.0) | <0.001 |
| Durable floor (%)  No  Yes | 175 (39.0)  270 (60.1) | 163 (46.4)  186 (53.0) | 0.037 |
| Durable roof (%)  No  Yes | 19 (4.2)  428 (95.3) | 89 (25.4)  262 (74.6) | <0.001 |
| Household death in last 12 months (%)  No  Yes | 400 (89.1)  49 (10.9) | 291 (82.9)  60 (17.1) | 0.011 |
| Perceived barriers to providing first aid during a health emergency (%)  No  Yes | 74 (16.5)  289 (64.4) | 12 (3.4)  316 (90.0) | <0.001 |
