# Supplementary material for: Seeking and reaching emergency care: A cross sectional household survey across two Liberian counties
Source: PLOS Glob Public Health. 2023 Nov 20;3(11):e0002629. doi: 10.1371/journal.pgph.0002629 (PMC10659191; doi:10.1371/journal.pgph.0002629)
Supplement: S2 Table — (DOCX) [file pgph.0002629.s003.docx]

S2 Table. Univariable and multivariable characteristics associated with facility-based emergency care utilization in the 12 months prior to survey, among residents in and around Monrovia, Montserrado County (N=399).

| Characteristic | Unadjusted OR (95% CI) | Adjusted OR (95% CI)* |
| --- | --- | --- |
| Perceived barriers to providing first aid during a health emergency | 9.43 (4.91-18.13) | 6.57 (3.33-12.96) |
| Non-English speaking | 6.13 (3.55-10.56) | 3.38 (1.83-6.26) |
| Low income | 2.27 (1.36-3.77) | 2.54 (1.40-4.61) |
| No electricity | 3.91 (2.19-6.97) | 1.84 (0.94-3.62) |
| Non-durable roof | 4.38 (0.49-39.56) | -- |
| No latrine | 2.76 (0.53-14.37) | -- |
| Death 12 months | 1.46 (0.79-2.72) | -- |

AUC=0.7623
