# Supplementary material for: Seeking and reaching emergency care: A cross sectional household survey across two Liberian counties
Source: PLOS Glob Public Health. 2023 Nov 20;3(11):e0002629. doi: 10.1371/journal.pgph.0002629 (PMC10659191; doi:10.1371/journal.pgph.0002629)
Supplement: S3 Table — (DOCX) [file pgph.0002629.s004.docx]

S3 Table. Univariable and multivariable characteristics associated with facility-based emergency care utilization in the 12 months prior to survey, among residents of Lofa County (N=401).

| Characteristic | Unadjusted OR (95% CI) | Adjusted OR (95% CI)* |
| --- | --- | --- |
| Non-durable roof | 14.17 (7.99-25.11) | 12.60 (7.03-22.56) |
| No electricity | 5.91 (2.46-14.19) | 4.00 (1.55-10.31) |
| Death 12 months | 2.00 (1.16-3.44) | 2.08 (1.11-3.90) |
| No latrine | 3.33 (2.18-5.08) | -- |
| Non-English speaking | 1.60 (1.02-2.52) | -- |
| Low income | 0.96 (0.46-2.00) | -- |

AUC=0.7806
